# Effect of pretreatment with matrix metalloproteinase inhibitors on the durability of bond strength of fiber posts to radicular dentin

**DOI:** 10.1002/cre2.569

**Published:** 2022-06-20

**Authors:** Kiamehr Ghazvehi, Aida Saffarpour, Sareh Habibzadeh

**Affiliations:** ^1^ Department of Prosthodontics, School of Dentistry, International Campus Tehran University of Medical Sciences Tehran Iran; ^2^ Dental Research Center, Dentistry Research Institute Tehran University of Medical Sciences Tehran Iran; ^3^ Department of Operative Dentistry, School of Dentistry, International Campus Tehran University of Medical Sciences Tehran Iran

**Keywords:** chlorhexidine, dimethyl sulfoxide, fiber post, matrix metalloproteinase

## Abstract

**Objectives:**

Application of matrix metalloproteinases inhibitors has been suggested to improve the durability of resin–dentin bonding. The purpose of this study was to evaluate the effect of dimethyl sulfoxide (DMSO), carbodiimide (EDC), and chlorhexidine (CHX) treatment on the push‐out bond strength of fiber posts to radicular dentin.

**Materials and Methods:**

24 extracted premolars were assigned randomly to 4 groups after root canal treatment and post space preparation (*n* = 6). In the first, second, and third groups, radicular dentin was treated with 1 ml of 5% DMSO, 0.3 M EDC, and 2% CHX, respectively. The fourth group (control) received no treatment. The root canals were primed with ED primer II, and the fiber posts were cemented with Panavia F2.0. In each group, half of the specimens were subjected to the push‐out test and the other half to 3000 thermal cycles before testing. Data were analyzed using two‐way analysis of variance via SPSS version 20 (*p* < .05).

**Results:**

Among the nonthermocycled specimens, the values of push‐out bond strength were observed in the control, EDC, CHX, and DMSO groups, in decreasing order. Among the thermocycled specimens, the values were observed in the control, EDC, DMSO, and CHX groups in decreasing order. Thermocycling had a significant adverse effect on the push‐out bond strength (*p* = .015), but the effect of material (*p* = .375), and the interaction effect of material and thermocycling (*p* = .998) were not significant.

**Conclusions:**

Application of CHX, DMSO, and EDC had no significant effect on the bond strength of fiber posts to radicular dentin.

## 1 INTRODUCTION

Fiber‐reinforced posts are routinely used to restore endodontically treated teeth due to adequate retention, optimal esthetics, and having an elastic modulus comparable to that of dentin (Naumann et al., 2012). Nonetheless, achieving a strong bond between the resin cement and radicular dentin is challenging, and even if achieved, it undergoes degradation over time (Bitter & Kielbassa, 2007; Ferrari et al., 2000; Naumann et al., 2012). Water/oral fluid sorption, polymer swelling, resin leaching, and degeneration of the hybrid layer due to hydrolytic degradation of collagen fibrils activated by matrix metalloproteinases (MMPs) and cysteine cathepsins on the surface of radicular dentin is among the most essential mechanisms of bond deterioration (Dionysopoulos, 2016; Santos et al., 2009; Tay et al., 2006). Attempts have been made to enhance the quality and longevity of resin‐dentin bond strength, by increasing the resistance of collagen against enzymatic degradation. Dentin biomodifiers, such as MMP inhibitors (either endogenous or exogenous) and collagen cross‐linkers, have been used to pretreat the demineralized dentin or incorporated into the bonding components (Mazzoni et al., 2018; Singh et al., 2017).

Chlorhexidine (CHX), as a nonspecific protease inhibitor, has been shown to maintain the hybrid layer through binding to carboxyl and hydroxyl groups of collagen and phosphate groups in hydroxyapatite crystallites in mineralized dentin (Gunaydin et al., 2016). Therefore, the application of CHX on etched dentin is recently increasing (Liu et al., 2011; Scaffa et al., 2012). Controversies yet remain, and the effectiveness of the results of other studies needs to be verified (Bitter et al., 2014; Leitune et al., 2010). Ricci et al. (2010) stated that water solubility and reversibility of the electrostatic bond of CHX to collagen results in the MMP inhibition property of CHX decreasing gradually within 18–24 months.

Carbodiimide, namely 1‐ethyl‐3‐(3‐dimethylaminopropyl) carbodiimide (EDC), is another MMP inhibitor, recently introduced as a nonspecific protein cross‐linker with low toxicity (Liu et al., 2011; Scheffel et al., 2015). It has been proposed that activation of carboxylic groups and cross‐linking of collagen fibrils by EDC preserve the dentin–resin bond mechanical properties (Liu et al., 2011; Tezvergil‐Mutluay et al., 2012). Various EDC solutions have been investigated to find a protocol of EDC dentin surface coating that best improves the bond strength over time; however, little attention has been paid to radicular dentin (Mazzoni, Angeloni et al., 2013; Mazzoni et al., 2018; Saffarpour et al., 2020; Tang et al., 2016).

Dimethyl sulfoxide (DMSO: (CH_3_)_2_SO) is a solvent (with a highly polar S═O group and two hydrophobic CH_3_ groups), with enhanced penetration into biological surfaces through dissociation of the extracellular collagen matrix. Its mechanism is said to either compete with water molecules in interpeptide hydrogen, inhibiting the interpeptide hydrogen bonds in the collagen matrix, or alter the collagen interfibrillar network (Tezvergil‐Mutluay et al., 2012). Therefore DMSO may serve as an attractive candidate to solvate adhesive constituents as well dental monomers (Mazzoni, Angeloni et al., 2013). Recently, it was reported that the aqueous solution of DMSO can effectively improve the immediate and long‐term bond strength and nanoleakage of adhesives in concentrations of 0.01%–20% through the integration of the hybrid layer (Salim Al‐Ani et al., 2018). However, its possible effectiveness for pretreatment of dentin before fiber post cementation has yet to be investigated.

Although recent literature has recently paid attention to the effects of dentin pretreatment with MMP inhibitors on the bond strength of various adhesive systems, few studies have evaluated their comparative effects, and limited data is found on radicular dentin (Saffarpour et al., 2020). Thus, this study sought to assess and compare the effects of DMSO, EDC, and CHX on the push‐out bond strength of fiber posts cemented with self‐etch (SE) resin cement to radicular dentin. The null hypothesis was that radicular dentin pretreatment with various MMP inhibitors does not improve the immediate or long‐term dentin bond strength of fiber posts after thermocycling.

## 2 MATERIALS AND METHODS

This in vitro experimental study was performed on 24 human premolars, extracted for orthodontic reasons at the department of maxillofacial surgery of Tehran University of Medical Sciences, School of Dentistry, International Campus, after informed consent was obtained under a protocol approved by the ethics committee of Tehran University of Medical Sciences (IR.TUMS.DENTISTRY.REC.1397.124). Periapical radiographs were taken to ensure the presence of single canals, absence of root calcifications, and closed apices. Teeth with more than one root canal, visible cracks, fractures, caries, anomalies or defects, severe wear, hypoplasia, or decalcification were excluded and replaced. The selected teeth were cleaned from debris, calculus, and tissue residues by an ultrasonic scaler and prophylaxis paste (DentaFlux, Madrid, Spain) and immersed in 0.5% chloramine T solution (Merck KGaA, Darmstadt, Germany) for disinfection. The teeth were then stored at 100% humidity in saline, incubated at 37 in labeled containers, and utilized within 1 month of extraction.

### 2.1 Preparation of the teeth

The roots were separated at the cementoenamel junction from the crowns by a diamond saw (D&Z, Berlin, Germany) underwater irrigation such that the remaining root length was 15 mm. The root canals were prepared with endodontic K‐files (Dentsply Maillefer, Ballaigues, Switzerland) under irrigation with 2.5% sodium hypochlorite up to #40 master apical file at a working length 1 mm short of the apex. The root canals were cleaned and shaped up to file #80 using the step‐back technique, according to the standard protocol. Next, the roots were obturated with gutta‐percha (Aria Dent, Asia Chemi Teb, Tehran, Iran) and AH26 sealer (Dentsply Sirona, PA, USA) using the lateral condensation technique. The optimal quality of obturation was later confirmed by periapical radiographs. The coronal part of the roots was sealed with light‐cure glass ionomer cement (Fuji II LC; GC Corporation, Tokyo, Japan) (Shafiei, Memarpour et al., 2016; Shafiei, Yousefipour et al., 2016). The roots were then immersed in distilled water at room temperature for 1 week to allow the complete set of the sealer. Next, a 10‐mm depth post space was prepared using an appropriate size of peeso reamer (Dentsply Sirona, PA, USA) and Exatec‐S drill (Hahnenkratt, Germany) of the fiber post. Periapical radiographs were obtained to ensure the complete removal of gutta‐percha from the root canals. Next, the fiber posts were tried in the canals, and the canals were cleaned with ethanol and dried with paper points (Meta Biomed, Chalfont, USA). The root canal space was rinsed with 1 ml of 18% ethylenediaminetetraacetic acid (EDTA; Cerkamed, Stalowa Wola, Poland) for 1 min to eliminate debris and residual filling materials, followed by a final rinse of water for 1 min and then dried (Shafiei, Yousefipour et al., 2016). The teeth were then randomly divided into four groups as follows:

Group 1: Radicular dentin was rinsed with 1 ml of 5% aqueous solution of DMSO (Thermo Fisher, MA, USA) for 60 s and blotted dry.

Group 2: Radicular dentin was rinsed with 1 ml of 0.3 M EDC (Thermo Fisher, MA, USA) for 60 s and blotted dry.

Group 3: A micro brush was used to apply 1 ml of 2% CHX digluconate solution (GLUCO‐CHeX‐ Cerkamed, Stalowa Wola, Poland) to the root canal walls for 60 s and blotted dry.

Group 4: This group served as the control group and did not receive any intervention.

### 2.2 Cementation process

After the roots were dried with air spray for 20 s, canals were primed with ED Primer II (SE; ED Primer) for 30 s. Next, the fiber posts (Exacto, Angelus, Londrina, PR‐Brazil) were placed in the canals with gentle finger pressure and cemented with Panavia F2.0 SE resin cement (Kuraray, Osaka, Japan) according to the manufacturer's instruction. After removing the excess cement, it was cured for 40 s (VIP Junior; Bisco, Schaumburg, IL, USA) with a light intensity of 600 mW/cm^2^. Light‐cure glass ionomer (Fuji II LC, GC, Tokyo, Japan) was used to seal the canals coronally. The specimens were stored in 100% humidity at room temperature for 24 h. Next, the teeth were mounted in acrylic blocks (Pekatray, Baye, Leverkuser, Germany) for the push‐out test.

### 2.3 Push‐out test

Half of the specimens in all four groups were sectioned into six 1‐mm‐thick slices by a cutting machine (Mecatome T201 A, Presi Grenoble, France). The first coronal section was not included and the remaining 15 slices were submitted to a compressive load at a crosshead speed of 0.5 mm/min (Shafiei, Yousefipour et al., 2016). The load was applied to the center of the posts apicocoronally by a universal testing machine (OZ50; Zwick, Germany). Loading was continued until the post was dislodged. The peak force value was recorded in Newton (N) and the push‐out bond strength in MPa was calculated by the following formula:

$$\text{Push}-\text{out}\;\text{bond}\;\text{strength}=\frac{F}{A}=\frac{\mathrm{maximum}\;\mathrm{loaded}\;\mathrm{force}}{\mathrm{JI}\left(R1+R2\right)\sqrt{{\left(R1-R2\right)}^{2}}+h^{2}},$$

where R1 is the radius of the post at the coronal side of the specimen,  R2 is the radius of the post at the apical side of the specimen, and  h is the height of the slice.

The other half of the roots in each group underwent thermocycling (TC‐300; Vafaei, Industrial, Iran). A total of 3000 thermal cycles (Ghavami‐Lahiji et al., 2018; Morresi et al., 2014) were applied between 5°C and 55°C with a dwell time of 30 s and a transfer time of 15 s. The rest of the procedures resembled the first half group and sections obtained in each group underwent the push‐out test. Data were analyzed using SPSS version 20 and reported using descriptive statistics via two‐way ANOVA (*p* < .05).

## 3 RESULTS

**Figure 1:**
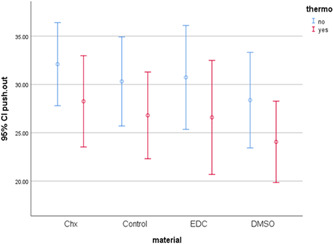
Error bar of the mean and 95% confidence interval of push‐out bond strength (MPa) of fiber posts to radicular dentin before and after thermocycling. CHX, chlorhexidine; CI, confidence interval; DMSO, dimethyl sulfoxide; EDC, carbodiimide.

Table 1 and Figure 1 show the mean values for push‐out bond strength of fiber posts to radicular dentin using CHX, DMSO, and EDC, with or without thermocycling. In the non‐thermocycled specimens, the maximum push‐out bond strength was noted in the CHX group followed by EDC, control, and DMSO groups. In the thermocycled specimens, the maximum push‐out bond strength of fiber posts to radicular dentin was noted in CHX, control, EDC, and DMSO groups, in decreasing order. According to a two‐way analysis of variance (ANOVA), thermocycling adversely affected the push‐out bond strength of fiber posts to radicular dentin (*p* = .015). However, the effect of material type (*p* = .375) and the interaction effect of thermocycling and material (*p* = .998) on push‐out bond strength was not significant. Therefore, no significant difference was noted in the push‐out bond strength while using CHX, DMSO, and EDC compared with the controls (*p* = .686).

**Table 1:** Push‐out bond strength of fiber posts to radicular dentin before and after thermocycling

Material	Thermocycling	Minimum	Maximum	Mean	Std. deviation
CHX	No	20.82	46.04	32.09	7.768
	Yes	12.29	43.79	28.25	8.529
Control	No	14.31	41.02	30.30	8.321
	Yes	16.59	50.17	26.79	8.101
EDC	No	13.41	51.12	30.72	10.095
	Yes	11.12	41.74	26.58	10.223
DMSO	No	14.09	44.85	28.37	8.932
	Yes	11.86	36.85	24.05	7.607

Abbreviations: CHX, chlorhexidine; DMSO, dimethyl sulfoxide; EDC, carbodiimide.

## 4 DISCUSSION

Providing a durable bond between the cement and radicular dentin is essential for adequate retention of fiber posts and subsequent success of the restoration. MMPs and cysteine cathepsins are involved in the degradation of collagen fibers in partially resin‐infiltrated hybrid layers. Thus, it appears that the application of MMP inhibitors before dentin adhesives may be a logical step to increase the durability of the hybrid layer (Victorino et al., 2016). In this study application of CHX, EDC, and DMSO on push‐out bond strength of fiber posts cemented to radicular dentin with SE resin cement, yielded no significant difference compared with the controls.

CHX effectively inhibits MMP‐2, MMP‐8, MMP‐9, and cysteine cathepsins (Correa et al., 2016). The concentration of CHX used for inhibition of MMPs in studies is usually 2% (Bitter et al., 2014; Correa et al., 2016; Wang et al., 2013). The effect of CHX on bond strength is still a matter of debate. Victorino et al. (2016), Cecchin et al. (2011a, 2011b, 2014), and Wang et al. (2013) showed that pretreatment of radicular dentin with CHX did not interfere with the immediate bond strength of fiber posts cemented with resin cements [25,27–30]. These results however were not admitted by Gunaydin et al. (2016), Campos et al. (2009), Lenzi et al. (2012), and Boruziniat et al. (2013) in similar studies. A recent systematic review by Dionysopoulos (2016) also failed to reveal a clear effect of CHX on the longevity of dentin bonds. The type and concentration of the applied solution, the type and composition of the adhesive system, the dentinal substrate (coronal vs. radicular and sound vs. caries‐affected dentin), and other factors related to the study design are all responsible for the diversity of results. In the current study, the mean values for push‐out bond strength in the CHX group were the highest among all, although the difference was not significant.

It has been reported that human gelatinases are inactivated by DMSO. Increased penetration of adhesives into the collagen matrix and wetting of collagens which occurs by applying this solvent, has been shown to prevent the degradation of the resin–dentin bond after 6 and 12 months (Tjaderhane et al., 2013). DMSO may also reduce the number of water molecules entrapped between the polymeric chains, thus can break down the self‐associative tendency of water (Tjaderhane et al., 2013). Salim Al‐An et al. (2018) demonstrated that pretreatment of dentin with DMSO as a primer at 1%–5% concentrations could improve the integrity of the resin–dentin interface. In a study by Shafiei, Memarpour et al. (2016), dentin pretreatment with DMSO resulted in prolonging the bond strength of fiber posts cemented adhesively with an etch‐and‐rinse (E&R) system. The same results were achieved by Tjaderhane et al. (2013) after 6 and 12 months. The current study, however, failed to reveal a positive effect for pretreatment of dentin using 5% DMSO and an SE adhesive system. Overall, there is limited information regarding the effect of DMSO pretreatment on dentin bond strength, especially radicular dentin. The controversy in results could be attributed to the type of adhesives used. Sharaffedin et al. (2016) commented that the application of DMSO on surface dentin using a total‐etch system had a much more profound positive effect on resin–dentin interface than deep dentin and using an SE adhesive system. A recent study by Mello et al. (2022) also stated that although dentin pretreatments containing DMSO theoretically improve the encapsulation of collagen fibers as well as the quality of the collagen–resin interface, they do not actually improve the bonding of universal adhesives in E&R or SE modes due to the formation of residual moisture, which impairs volatilization of the DMSO solvent.

Little attention has been paid to the beneficial effects of EDC on dentin bond durability, and a clear protocol of EDC dentin surface coating is still under investigation (Shafiei, Yousefipour et al., 2016; Tang et al., 2016). Shafiei, Yousefipour et al. (2016) reported that the EDC could diminish the effect of aging on the bond strength of fiber posts to radicular dentin using three types of resin cement. The demineralized dentin cross‐link degree was shown to increase by EDC compared with control and ethanol treatments in a study by Tang et al. (2016). In agreement with the current study, Comba et al. (2019) reported that the application of EDC did not cause a significant change in bond strength of adhesive‐cemented fiber posts after 24 h; however, it maintained the bond strength after 1 year of storage in artificial saliva, compared with the controls. Evaluating the resin‐dentin interface pretreated with EDC after 1 year of aging with SE and E&R adhesive systems, the bond strength was stabilized in a study by Mazzoni et al. (2018).

Clinical trials are time‐consuming and costly; therefore, in vitro simulation is often performed to assess the effect of aging on materials (Gale & Darvell, 1999). A total of 10,000 thermal cycles are often used to simulate 1 year of clinical service (Gale & Darvell, 1999). Thermocycling is believed to decrease the bond strength of enamel and dentin (Daneshkazemi et al., 2013; Sirisha et al., 2014; Xie et al., 2010). The current results also implied the negative effect of thermocycling on the push‐out bond strength of fiber posts to radicular dentin in all experimental groups. Failure in adhesion can be due to hydrolytic degradation of adhesives or degradation of collagen fibers in the hybrid layer by internal enzymes (Conte et al., 2019). Thus, it appears that the application of MMP inhibitors could not compensate for the bond strength reduction caused by thermocycling in the current study. Bitter et al. (2014) reported that thermocycling had no significant effect on the push‐out bond strength of fiber posts to radicular dentin (Bitter et al., 2014). In the present study, 3000 thermal cycles were applied, corresponding to approximately 3 months of clinical service. Assessment of the results of similar studies indicates that MMP inhibitors increase the long‐term durability of bonds, and their application may not cause a significant short‐term change (Cecchin et al., 2011a, 2011b, 2014). A recent systematic review on the role of CHX as an MMP inhibitor on dentin bonding also stated that its use has no significant effect on immediate resin–dentin bond strength, while after aging for 6, 12, and 24 months, better bond strength has been achieved with CHX compared to the controls (Kiuru et al., 2021). Thus, a lack of significant effect on the bond strength in the present study may be due to the short duration of aging. On the other hand, the use of thermocycling for artificial aging is a matter of debate as there is no definite evidence of fatigue due to thermal stresses (Gale & Darvell, 1999). Other methods, such as immersion in artificial saliva for a certain period, may lead to more realistic results (Shafiei, Yousefipour et al., 2016).

In the present study, the root canal space was rinsed with EDTA before cementation to eliminate the second thick smear layer and expose the dentinal tubules allowing the SE cement and SE primer to adequately penetrate the radicular dentin (Shafiei, Memarpour et al., 2016). EDTA can adequately disinfect the dentin and allow the dissociation of exposed collagen. Evidence shows that EDTA inhibits MMPs (Shafiei, Memarpour et al., 2016). Therefore, copious water irrigation was performed during the bonding process to ensure there was no residual EDTA to inhibit the activity of MMPs.

Literature provides us with conflicting results regarding the effect of adhesive type on the durability of fiber posts bond strength in root dentin in the presence of MMP inhibitors. In the present study, the SE adhesive system was used. Bitter et al. (2013) reported that bond strength values obtained with this system are higher than that of two‐step E&R or self‐adhesives (SA). More rapid destruction of the hybrid layer has been reported for three‐step E&R adhesives compared with mild two‐step SE ones (Zheng et al., 2015). Mazzoni, Scaffa, et al. (2013) attributed this to the higher levels of MMP‐2 and MMP‐9 activity demonstrated for E&R adhesives, which autodegrades the collagen matrices in the hybrid layer, created by the dentin bonding systems. In a study by Shafiei, Yousefipour et al. (2016) evaluating the durability of adhesively cemented fiber posts in root canals using three adhesive cements, SA cement exhibited the highest immediate strength. Limitations regarding the difficulty in the adhesion procedure, such as homogeneous etching, complete acid washing, optimal dentin wetness, complete resin penetration, uniform application, and solvent evaporation from the primer/adhesive in narrow and deep root canal spaces with limited access, were attributed for the lower immediate bond strength of E&R cement especially. These authors, however, did not find any significant differences in the bond strength of the three cements used after aging. This was further explained by the relatively high hydrophilic acidic nature of SA cement, its water sorption, and the resulting hydrolytic degradation of the SA‐radicular dentin interface during storage. Activation of MMPs and cathepsins during SA bonding procedures, which occur due to the low primary pH of the residual unstable acidic monomers in them, may lead to the degradation of coronal or radicular dentin collagen.

Corroborated in other studies, higher microtensile bond strength values have been reported using the E&R strategy both immediately and after aging. The micromorphology of the interface shows the presence of longer tags and a thicker hybrid layer since the acid conditioning increases the surface energy and removes the smear layer (Dačić et al., 2021; Wagner et al., 2014). Stape et al. (2018) suggested that recognizing the use of E&R leaves more residual water in the hybrid layer than does the SE strategy, the use of pretreatments, whether solubilized in water or ethanol or not solubilized, could lead to the replacement or displacement of residual water more effectively, in a relatively short application period. Comparing the effect of DMSO pretreatment in E&R or SE modes, Mello et al. (2022) found that the interaction between treatments, storage time, and etching modes was not significant for microtensile bond strength; however, aging decreased the bond strength over time only for the E&R strategy.

The push‐out test is a reliable technique for the assessment of the bond strength of fiber posts to radicular dentin. Nonetheless, it should be noted that the application of push‐out forces to fiber posts is less compared to the application of actual functional loads to fiber posts in a clinical setting (Bitter et al., 2014). The microtensile test has higher accuracy than the push‐out test. However, specimen preparation for the microtensile test is difficult, and many specimens break before testing. Data dispersion is also high in microtensile tests (Amin Salehi & Shapoor Falsafi, 2008).

This article failed to report the mode of failure and its analysis in study groups. The authors suggest future studies with a larger sample size, use of other bonding systems rather than the SE, prolonged aging in the form of higher thermal cycles, or other forms of artificial aging. Evaluation of the bond strength after final restoration placement, which could simulate a closer clinical situation to confirm the potential benefits of MMP inhibitors, is also suggested.

## 5 CONCLUSION

The current results revealed that the application of CHX, DMSO, and EDC had no significant effect on the push‐out bond strength of fiber posts to radicular dentin using an SE bonding system. Thermocycling negatively affected the push‐out bond strength of fiber posts to radicular dentin.

## AUTHOR CONTRIBUTIONS

*Carrying out the experiments, collection of data; data analysis/interpretation*: Kiamehr Ghazvehi. *Contribution to the study design*: Aida Saffarpour. *Study design; planning and supervision of the work, writing of the manuscript; proofreading the manuscript*: Sareh Habibzadeh.

## CONFLICTS OF INTEREST

The authors declare no conflicts of interest.

## Data Availability

The raw data and processed data for this article are available via an excel file.
